# Supplementary material for: Proposal of the Implementation Theory Selection Model and exemplar application in fall injury prevention
Source: PLoS One. 2024 Nov 27;19(11):e0310117. doi: 10.1371/journal.pone.0310117 (PMC11602108; doi:10.1371/journal.pone.0310117)
Supplement: S1 File — (PDF) [file pone.0310117.s001.pdf]

A SYSTEMATIC APPROACH TO SELECT THEORIES, MODELS AND FRAMEWORKS TO UNDERPIN THE STUDY OF IMPLEMENTING HIP PROTECTORS IN LONG-TERM CARE

BOX 1: Administrative information.

Initials (First, Last):	
Date (YYYY-MM-DD):	
TMF ID (XXX):	
TMF Name:	

BOX 2: Information about seminal source(s) reviewed to appraise TMF.

Seminal Source #1 ID (XXX-X, XXX-XX)	
Seminal Source #2 ID (XXX-X, XXX-XX)	
Seminal Source #3 ID (XXX-X, XXX-XX)	
Seminal Source #4 ID (XXX-X, XXX-XX)	

BOX 3: Supplementary search for seminal sources (optional). Please note you can insert more rows if necessary.

Reference #1<br>First Author (Last Name):	
Year:	
Title:	
Source (website, PubMed, etc.):	
Type (journal article, etc.):	
Reference #2<br>First Author (Last Name):	
Year:	
Title:	
Source (website, PubMed, etc.):	
Type (journal article, etc.):	

BOX 4: Summary of construct(s)[1] contained in TMF, analytic level of TMF (e.g., individual, organization, community, etc.), and other relevant details.

[1]Note that the term 'constructs' is used broadly to refer to the types of variables (also known as classes or domains), as well as specific variables within a category, class or domain that are contained in the TMF.

Construct(s):	
Analytic level(s):	
Other details:	

BOX 5: Extent of agreement or disagreement with statements regarding the fit of the TMF to our project and study aims.

Statement	Rating (1-5)	Notes to explain your rating
TMF includes relevant constructs[1] (e.g., readiness; any other constructs you perceive are relevant).		•

[1]Note that the term ‘constructs’ is used broadly to refer to the types of variables (also known as classes or domains), as well as specific variables within a category, class or domain that are contained in the TMF.		
TMF addresses one or more relevant analytic levels (e.g., individual; organization).		•
TMF provides an explanation of how included constructs influence implementation and/or each other.		•
TMF has a clear and useful figure or table depicting included constructs and relationships among them.		•
TMF provides a step-by-step approach for applying it.		•
TMF focuses on a relevant implementation outcome (e.g., acceptability; adoption; appropriateness; feasibility; fidelity; implementation cost; penetration; sustainability).		•
TMF includes meaningful, face-valid explanations of proposed relationships and constructs.		•
TMF provides methods for promoting implementation in practice.		•
TMF proposes testable hypotheses.		•
TMF contributes to an evidence base and/or theory development because it has been used in empirical studies.		•
TMF is generalizable to our context.		•
TMF is generalizable to other disciplines and/or contexts.		•

BOX 6: Overall perceived fit of the TMF with each of our study objectives.

Objective	Rating (0-2)	Notes to explain your rating
**1a**		•
**1b**		•

BOX 7: Additional feedback or comments about the TMF.

**Additional feedback:**	•
