# Supplementary material for: Proposal of the Implementation Theory Selection Model and exemplar application in fall injury prevention
Source: PLoS One. 2024 Nov 27;19(11):e0310117. doi: 10.1371/journal.pone.0310117 (PMC11602108; doi:10.1371/journal.pone.0310117)
Supplement: S2 File — (PDF) [file pone.0310117.s002.pdf]

PRACTICAL, ROBUST IMPLEMENTATION AND SUSTAINABILITY (PRISM) MODEL

Summary statement:

- The PRISM Model evaluates how the health care program or intervention interacts with the recipients to influence program adoption, implementation, maintenance, reach, and effectiveness.
- The outcome measures are guided by an evaluation framework known as the RE-AIM framework.
- Implementation success is dependent on how the elements of a specific program or intervention are perceived by the organization and staff to be targeted, as well as patients.
- Organizations have characteristics that affect their ability to successfully change behaviors in a given clinical area, which need to be considered at three organizational levels: top management, middle managers and frontline teams.
- Characteristics of patient recipients of interventions need to be considered to maximize intervention effectiveness and reach important patient subgroups.
- Successful implementation requires a carefully crafted infrastructure.
- Elements relevant to the external environment may be some of the most powerful predictors of success and therefore are key to implementation and maintenance.

Graphical illustration:

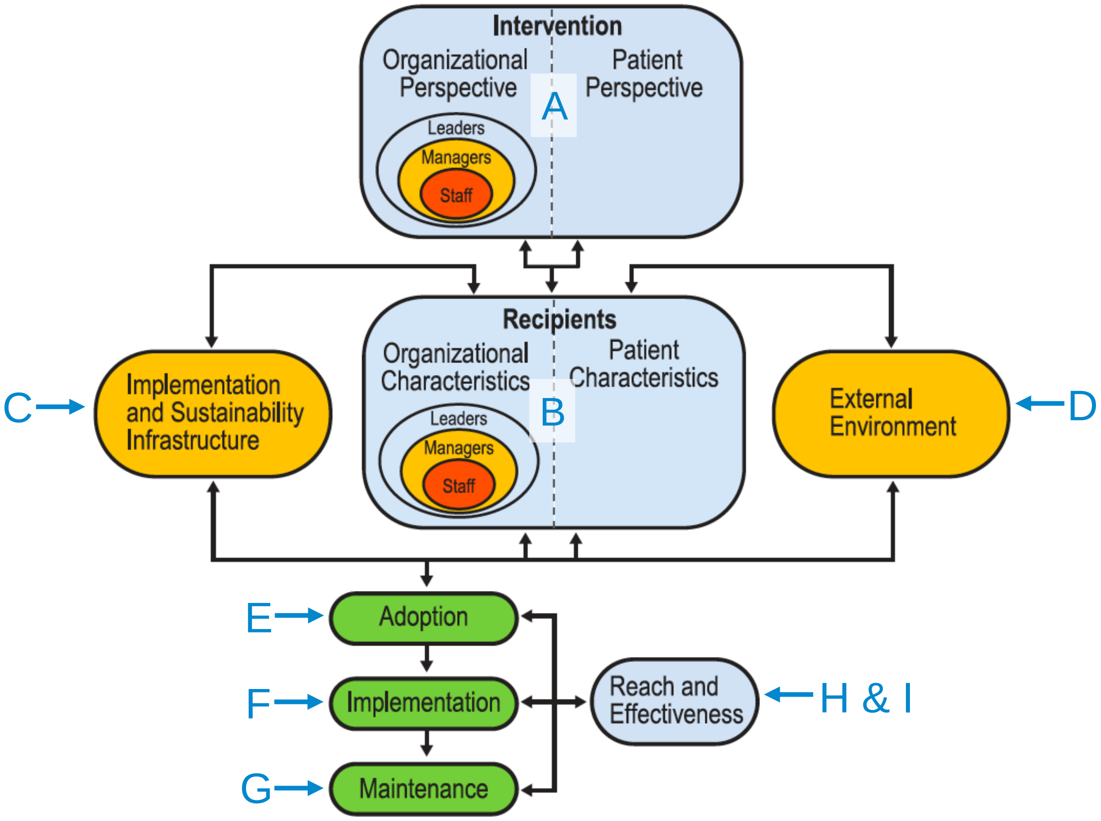

Key components (labelled in the figure above):

A. **Intervention** elements to consider from the ***organizational perspective*** include: readiness; strength of the evidence base; addresses barriers of frontline staff; coordination across departments and specialities; burden (complexity and cost); usability and adaptability; trialability and reversibility; ability to observe results. **Intervention** elements to consider from the ***patient perspective*** include:

patient centredness; provides patient choices; addresses patient barriers; seamlessness of transition between program elements; service and access; burden (complexity and cost); feedback of results.

B. **Recipients** elements to consider related to ***organizational characteristics*** include: organizational health and culture; management support and communication; shared goals and cooperation; clinical leadership; systems and training; data and decision support; staffing and incentives; expectation of sustainability. **Recipients** elements to consider related to ***patient characteristics*** include: demographics; disease burden; competing demands; knowledge and beliefs.

C. **Implementation and sustainability infrastructure** elements to consider include: performance data; sustainability infrastructure; dedicated team; adopter training and support; relationship and communication with adopters (bridge researchers); adaptable protocols and procedures; facilitation of sharing of best practices; plan for sustainability.

D. **External environmen**t elements to consider include: payor satisfaction; competition; regulatory environment; reimbursement; community resources.

E. **Adoption** = The absolute number, proportion, and representativeness of settings and intervention agents (people who deliver the program) who are willing to initiate a program, and why.

F. **Implementation** = At the setting level, implementation refers to the intervention agents' (people who deliver the program) fidelity to the various elements of an intervention's key functions or components, including consistency of delivery as intended, adaptations made to interventions and implementation strategies, and the time and cost of the intervention.

G. **Maintenance** = At the setting level, the extent to which a program or policy becomes institutionalized or part of the routine organizational practices and policies. At the individual level, the long-term effects of a program on outcomes after a program is completed.

H. **Reach** = The absolute number, proportion, and representativeness of individuals who are willing to participate in a given initiative, intervention, or program, and reasons why or why not.

I. **Effectiveness** = The impact of an intervention on important individual outcomes, including potential negative effects, and broader impact including quality of life and economic outcomes; and variability across subgroups.

Year first published: 2008

Estimated # citations: 180

Example applications:

- Implementing a breast cancer screening and navigation program for women from under-resourced communities
- Evaluating a multicomponent program to improve hypertension control in Guatemala
- Implementing and disseminating of a transition of care program for rural veterans
- Implementing a population-based colorectal cancer screening program

Website: N/A

Appraiser ratings:

Objective	Mean fit (range: 0-2[a])	Mean usability (range: 1-5[b])	Mean testability (range: 1-5[b])	Mean applicability (range: 1-5[b])
1a	2	4.3	3.7	4.3
1b	1.5			

[a]0=poor fit, 1=moderate fit, 2=good fit; [b]1=strongly disagree, 2=somewhat disagree, 3=neither disagree nor agree, 4=somewhat agree, 5=strongly agree
