# Supplementary material for: Proposal of the Implementation Theory Selection Model and exemplar application in fall injury prevention
Source: PLoS One. 2024 Nov 27;19(11):e0310117. doi: 10.1371/journal.pone.0310117 (PMC11602108; doi:10.1371/journal.pone.0310117)
Supplement: S3 File — (PDF) [file pone.0310117.s003.pdf]

THEORY, MODEL AND/OR FRAMEWORK (TMF) SELECTION MEETING AGENDA

Time	Duration	Topic	Speakers/Facilitators
**8:55-9:10**	**15 min**	**1. Welcome**	
8:55-9:02	7 min	order, welcoming remarks	K. Sibley; F. Hoekstra
9:02-9:10	8 min	Review agenda, process and “ground rules” for group discussion*	F. Hoekstra
**9:10-9:45**	**35 min**	**2. Round 1 consensus - objective 1a TMFs**	
9:10-9:18	8 min	Review and discuss objective 1a TMFs	A. Korall; All participants
9:18-9:33	15 min	Round 1 Talking Circle	All participants
9:33-9:40	7 min	Discuss feedback from Round 1 Talking Circle	All participants
9:40-9:45	5 min	Voting and re-voting, if necessary (possible break; TBD)	F. Hoekstra; M. Khan
**9:45-9:50**	**5 min**	**Break**	
**9:50-10:25**	**35 min**	**3. Round 2 consensus - objective 1b TMFs**	
9:50-9:58	8 min	Review and discuss objective 1b TMFs	A. Korall
9:58-10:13	15 min	Round 2 Talking Circle	All participants
10:13-10:20	7 min	Discuss feedback from Round 2 Talking Circle	All participants
10:20-10:25	5 min	Voting and re-voting, if necessary (possible break; TBD)	F. Hoekstra; M. Khan
**10:25-10:57**	**32 min**	**4. Round 3 consensus - final selection**	
10:25-10:30	5 min	Round 1 and Round 2 Results and silent generation of ideas (optional break)	F. Hoekstra
10:30-10:45	15 min	Round 3 Talking Circle	All participants
10:45-10:55	10 min	Discuss feedback from Round 3 Talking Circle	All participants
10:55-10:57	2 min	Voting	F. Hoekstra; M. Khan
**10:57-11:00**	**3 min**	**5. Conlusion and next steps**	
		Meeting outcome announced on or before June 10, 2021 and evaluation sent on May 27, 20	F. Hoekstra

*Sent out in advance on Wednesday, May 19, 2021
