# Supplementary material for: Proposal of the Implementation Theory Selection Model and exemplar application in fall injury prevention
Source: PLoS One. 2024 Nov 27;19(11):e0310117. doi: 10.1371/journal.pone.0310117 (PMC11602108; doi:10.1371/journal.pone.0310117)
Supplement: S4 File — (PDF) [file pone.0310117.s004.pdf]

Additional file 4: List of included and excluded theories, models and/or frameworks

Included theories, models and/or frameworks (n=23)

1. Active Implementation Framework
2. Behaviour Change Wheel (BCW)
3. Conceptual Model of Implementation Research
4. Consolidated Framework for Implementation Research (CFIR)
5. Critical Realism & the Arts Research Utilization Model (CRARUM)
6. Dissemination of Evidence-based Interventions to Prevent Obesity
7. Exploration, Preparation, Implementation and Sustainability (EPIS) Model
8. Implementation Drivers Framework
9. Implementation Effectiveness Model / Organizational Theory of Implementation of Innovations
10. Implementation of Change in Health Care
11. Interactive Systems Framework for Dissemination and Implementation
12. Knowledge Transfer and Exchange
13. Normalization Process Theory (NPT)
14. Organizational Readiness Theory (ORT)
15. Practical, Robust Implementation and Sustainability Model (PRISM)
16. Precede-Proceed Model
17. Promoting Action on Research Implementation in Health Services (PARiHS) framework
18. Push-Pull Capacity Model
19. Real World Dissemination
20. Research Development Dissemination and Utilization Framework

21. Social Marketing Framework
22. Sticky Knowledge
23. Theoretical Domains Framework (TDF)

Excluded theories, models and/or frameworks (n=43)

1. "4E" Framework for Knowledge Dissemination and Utilization
2. Action Research
3. Availability, Responsiveness & Continuity (ARC): An Organizational & Community Intervention Model
4. Caledonian Practice Development Model
5. Centers for Disease Control and Prevention (CDC) Division of HIV/AIDS prevention (DHAP) Research-to-Practice Framework
6. Community Based Participatory Research (CBPR)
7. Constructivist Theory (Piaget)
8. Continuous Quality Improvement (CQI)
9. Cooperation Theory
10. Davis' Pathman PRECEDE Model
11. Diffusion of Innovation Theory
12. Facilitating Adoption of Best Practices (FAB) Model
13. Five Step Public Health Approach
14. Four Level Model of Learning
15. Framework for the Transfer of Patient Safety Research into Practice
16. Group Dynamics Theory
17. Handbook of Implementation Science

18. Health Belief Model

19. Health Promotion Technology Transfer Process

20. Intervention Mapping

21. Knowledge to Action Framework

22. Pathways to Evidence Informed Policy

23. Positive Behavioral Interventions and Supports (PBIS)

24. Plan-Do-Study-Act Cycles (Deming)

25. Pragmatic-Explanatory Continuum Indicator Summary 2

26. Proctor’s Implementation Outcomes

27. Pronovost’s 4E Process Theory

28. Quality Enhancement Research Initiative (QUERI)

29. Reach, Effectiveness, Adoption, Implementation, Maintenance (RE-AIM) Framework

30. Replicating Effective Programs Plus Framework

31. Self-Determination Theory

32. Self-Efficacy Theory

33. Self-Regulation Theory

34. Six-Step Framework for International Physical Activity Dissemination

35. Social Cognitive Theory

36. Social Ecological Framework

37. Social Learning Theory

38. Technology Transfer Model

39. Theory of Cognitive Development (Piaget)

40. Theory of Organizational Change

41. Theory of Planned Behaviour

42. Transtheoretical Model of Behaviour Change

43. Utilization-Focused Surveillance Framework
